# The mitochondrial NADH pool is involved in hydrogen sulfide signaling and stimulation of aerobic glycolysis

**DOI:** 10.1016/j.jbc.2021.100736

**Published:** 2021-04-30

**Authors:** Victor Vitvitsky, Roshan Kumar, Marouane Libiad, Allison Maebius, Aaron P. Landry, Ruma Banerjee

**Affiliations:** Department of Biological Chemistry, Michigan Medicine, University of Michigan, Ann Arbor, Michigan, USA

**Keywords:** hydrogen sulfide, sulfide quinone oxidoreductase, aerobic glycolysis, electron transport chain, DMEM, Dulbecco's modified Eagle's medium, DPBS, modified PBS, ETC, electron transport chain, FBS, fetal bovine serum, *Lb*NOX, *Lactobacillus brevis* NADH oxidase, SQOR, sulfide quinone oxidoreductase, TCA, tricarboxylic acid

## Abstract

Hydrogen sulfide is synthesized by enzymes involved in sulfur metabolism and oxidized *via* a dedicated mitochondrial pathway that intersects with the electron transport chain at the level of complex III. Studies with H_2_S are challenging since it is volatile and also reacts with oxidized thiols in the culture medium, forming sulfane sulfur species. The half-life of exogenously added H_2_S to cultured cells is unknown. In this study, we first examined the half-life of exogenously added H_2_S to human colonic epithelial cells. In plate cultures, H_2_S disappeared with a t^1^/_2_ of 3 to 4 min at 37 °C with a small fraction being trapped as sulfane sulfur species. In suspension cultures, the rate of abiotic loss of H_2_S was slower, and we demonstrated that sulfide stimulated aerobic glycolysis, which was sensitive to the mitochondrial but not the cytoplasmic NADH pool. Oxidation of mitochondrial NADH using the genetically encoded mito-*Lb*NOX tool blunted the cellular sensitivity to sulfide-stimulated aerobic glycolysis and enhanced its oxidation to thiosulfate. In contrast, sulfide did not affect flux through the oxidative pentose phosphate pathway or the TCA cycle. Knockdown of sulfide quinone oxidoreductase, which commits H_2_S to oxidation, sensitized cells to sulfide-stimulated aerobic glycolysis. Finally, we observed that sulfide decreased ATP levels in cells. The dual potential of H_2_S to activate oxidative phosphorylation at low concentrations, but inhibit it at high concentrations, suggests that it might play a role in tuning electron flux and, therefore, cellular energy metabolism, particularly during cell proliferation.

In contrast to most quiescent cells that rely on the efficiency of oxidative phosphorylation to harvest energy from glucose catabolism, rapidly dividing cells dial up glycolytic flux in a process referred to as the Warburg effect (1). This metabolic switching is cued in part by growth factors and transforms mitochondria into central hubs for macromolecule precursor synthesis (2). In this metabolically reprogrammed setting, mitochondrial ATP generation becomes secondary to its role in anabolic precursor synthesis. The regulatory levers that dial down flux through the electron transport chain (ETC), however, continue to be elucidated.

H_2_S is a respiratory poison that targets complex IV (3). It is also an endogenously synthesized metabolite and a product of at least three enzymes in the sulfur metabolic network (4, 5). Two, cystathionine β-synthase and γ-cystathionase, are components of the transsulfuration pathway, which convert homocysteine to cysteine *via* cystathionine (6, 7). The third enzyme, mercaptopyruvate sulfurtransferase, resides in the catabolic arm of sulfur metabolism and converts 3-mercaptopyruvate to an enzyme-bound persulfide. The latter is transferred to a low-molecule-weight acceptor (*e.g.*, cysteine) or to a protein thiol (*e.g.*, on thioredoxin) from where H_2_S is subsequently eliminated (8, 9). Alternatively, the persulfide can potentially undergo further transsulfuration *via* transfer to other acceptors.

Quantitatively, the output of H_2_S *versus* cysteine from the transsulfuration pathway is unknown, and there is limited insight into how these competing catalytic activities are regulated (10). A dedicated sulfide oxidation pathway in the mitochondrion converts H_2_S to the largely innocuous products, thiosulfate and sulfate (Fig. 1) (11). The oxidation pathway begins with the conversion of H_2_S to a persulfide in a reaction catalyzed by the inner mitochondrial membrane protein, sulfide quinone oxidoreductase (SQOR) (12, 13). The electrons from this reaction are transferred *via* coenzyme Q to the ETC and enter at the level of complex III (14). The glutathione persulfide product of the SQOR reaction (13, 15, 16) is oxidized by ETHE1 (17) to sulfite, which is either oxidized to sulfate by sulfite oxidase or converted to thiosulfate by thiosulfate sulfurtransferase (or rhodanese) (18). Electrons from the sulfite oxidase reaction enter the ETC at the level of complex IV *via* cytochrome c (19).

**Figure 1:**
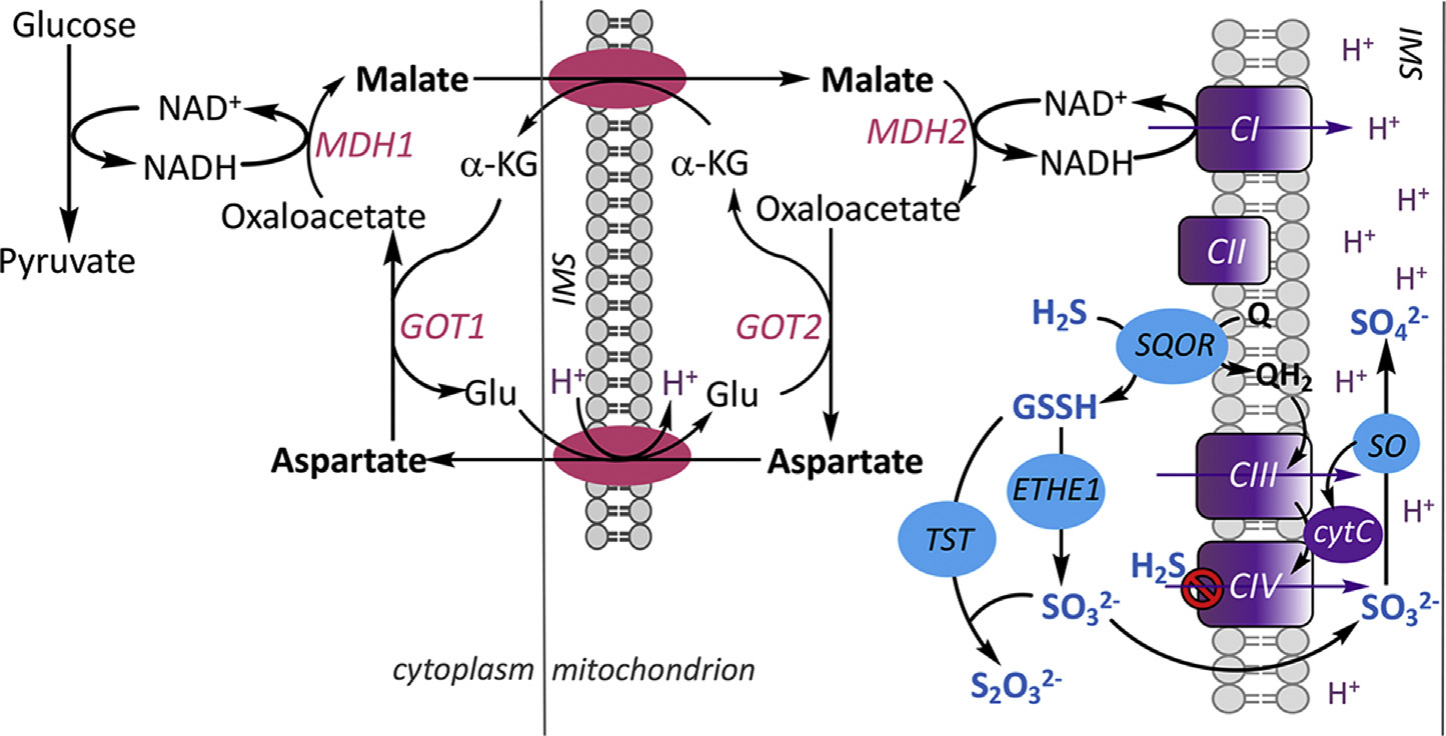
**Scheme showing the intersections between energy metabolism and H**_**2**_**S oxidation.** Reducing equivalents generated in the cytoplasm are moved to the mitochondrion *via* the malate–aspartate shuttle comprising a neutral α-ketoglutarate–malate and an electrogenic aspartate–glutamate transporter (*pink*). Conversion of malate to oxaloacetate in the mitochondrion regenerates NADH, which enters the electron transport chain (*purple*) at the level of complex I (CI). The *purple arrows* denote proton translocation. The sulfide oxidation pathway (*blue*) converts H_2_S to thiosulfate and sulfate with electrons from the oxidation catalyzed by SQOR entering at the level of complex III (CIII) and from sulfite oxidase (SO) at the level of complex IV (CIV). At higher concentrations, H_2_S inhibits CIV. α-KG, MDH1/2, GOT1/2, and IMS denote α-ketoglutarate, malate dehydrogenase 1 and 2, glutamate-oxoglutarate transaminase 1 and 2, and inter mitochondrial membrane space, respectively.

The physiological significance of endogenously produced H_2_S is underscored by the ubiquitous presence of the sulfide oxidation pathway. Based on the human protein atlas, the sulfide oxidation pathway enzymes are widely distributed rather than being restricted to epithelial tissue as might be expected for a detoxification pathway for dealing with environmental H_2_S. The mitochondrial location of this pathway, and its intersection with the ETC at two points, raises questions about whether its role is solely to detoxify H_2_S or potentially includes other functions, *e.g.*, ETC regulation.

H_2_S is redox active. It can react with oxidized cysteine thiols (*e.g.*, sulfenic acid or disulfides) to form persulfides or undergo oxidative metabolism to form reactive sulfur species (20, 21, 22). Much of the focus in the field of H_2_S redox signaling has been on protein persulfidation (23), a nonspecific oxidative modification of cysteines, which has been found on hundreds of proteins in proteomic studies (24, 25). Specific and quantitatively significant persulfidation that is correlated with the functional regulation of a target protein in a cellular setting remains to be demonstrated. It is likely that protein persulfidation is a stochastic marking reflecting local or global oxidation events (26). Although biosynthesis of a metabolite that functions as a respiratory poison presents a conundrum, its effect on complex IV remains the best characterized cellular effect of H_2_S to date. It is therefore instructive to consider H_2_S signaling in the context of redox metabolic programming that emanates from the ETC (26).

Studies examining the effects of H_2_S in cells typically involve its exogenous provision as a salt (*e.g.*, Na_2_S) or as a “pro-drug” (*e.g.*, GYY4137 (27)). Extracellular addition of H_2_S raises obvious concerns about physiological relevance, with the possible exception of epithelial cells such as colonocytes that are regularly exposed to high concentrations of H_2_S derived from microbial metabolism estimated to vary from 0.2 to 2.4 mM (28, 29). The volatility of H_2_S often requires protracted exposure to repeated doses (*e.g.*, 5–20 μM every 2 h for 5 days) to observe changes (30). Previously, we had observed the antiproliferative effect of H_2_S on malignant and nonmalignant colon epithelial cells by dosing with 100 or 300 μM Na_2_S every 12 h (31). Very little is known about the t^1^/_2_ of H_2_S under cell culture conditions, which is an important consideration due not only to its volatility but also to its ability to react with cystine and other oxidized thiols present in the culture medium.

We had previously screened six human colorectal carcinoma cell lines and shown that they exhibit higher expression levels of SQOR, ETHE1, and TST compared with a nonmalignant colon epithelial cell line (31). We had also demonstrated that exogenous H_2_S induced a reductive metabolic shift, decreasing the NAD^+^/NADH ratio and stimulating reductive carboxylation. Like other ETC poisons, H_2_S increases glycolysis, although the study reporting this effect concluded that it was mediated independently of its interaction with the ETC (30).

In this study, we demonstrate that the majority of H_2_S is rapidly lost (<30 min) from commonly used culture media (RPMI and Dulbecco's modified Eagle's medium [DMEM]) owing to its volatility, while a fraction is trapped as sulfane-sulfur species presumably *via* reaction with cystine and oxidized serum protein thiols. We report that H_2_S profoundly impacts energy metabolism and increases glucose consumption and lactate production, and that these changes are sensitive to the mitochondrial but not cytoplasmic NADH pool. Finally, we demonstrated that SQOR regulates these H_2_S-mediated cellular responses, revealing a previously unrecognized lever for controlling electron flux and coordinating energy metabolism across compartments.

## Results

### Kinetics of sulfide disappearance from cell culture medium

The sulfide salts Na_2_S and NaHS are widely used as exogenous sources of sulfide in cell culture experiments. H_2_S is volatile, and the kinetics of abiotic loss under standard culture conditions, which is expected to impact cellular responses, is largely unknown. We therefore examined the kinetics of sulfide loss under plate and suspension culture conditions that are routinely used in our laboratory, as a prelude to characterizing its effects on energy metabolism.

**Figure 2:**
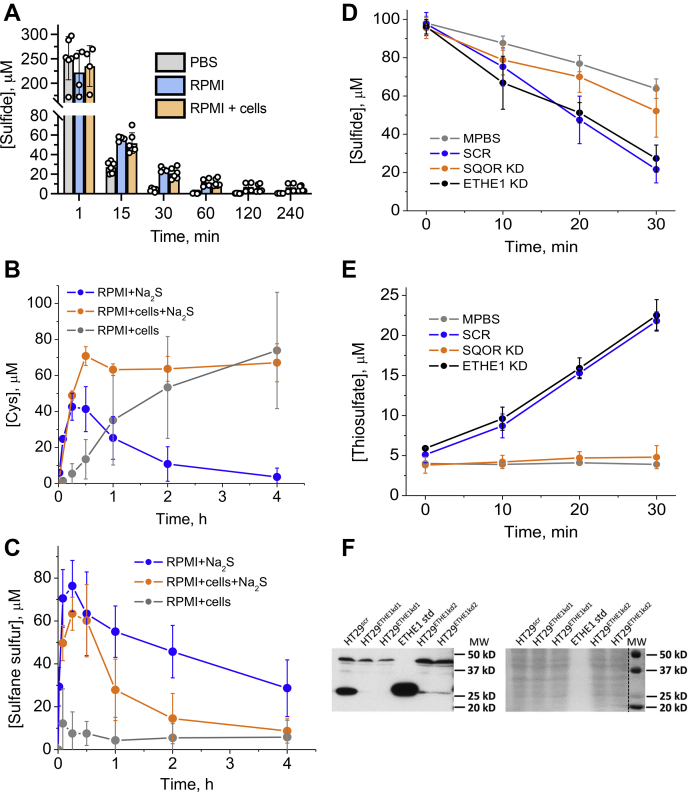
**Kinetics of sulfide disappearance under different experimental conditions.***A*, kinetics of Na_2_S (300 μM) disappearance from 6-cm culture plates containing PBS, or complete RPMI 1640 medium with or without HT29^scr^ cells. *B* and *C*, changes in the concentration of cysteine (*B*) and sulfane sulfur (*C*) in the same experiments as in *A* as well as in the presence of cells but absence of Na_2_S. *D*, kinetics of sulfide (100 μM) disappearance from modified PBS (DPBS) in the presence or absence of a 5% suspension (^w^/_v_) of HT29^scr^ cells or in the presence of HT29 cells with SQOR or ETHE1 knocked down. *E*, accumulation of thiosulfate in the experiments shown in (*D*). Data represent the mean ± SD of 3 to 5 experiments. *F*, Western blot (*left*) showing knockdown of ETHE1 in HT29 cells using two shRNA sequences and Ponceau S staining (*right*) for equal loading.

Sulfide addition to 6-cm plates containing PBS at 37 °C resulted in its rapid disappearance (Fig. 2*A*) with a t^1^/_2_ of ~3 min. Similarly, in RPMI 1640 cell culture medium supplemented with 10% fetal bovine serum, the sulfide concentration decreased 5- to 6-fold within 15 min and decreased with a t^1^/_2_ of ~4 min (Fig. 2*A*). Of interest, the sulfide concentration was maintained at ~5 to 10 μM in the RPMI medium for several hours, which was independent of whether or not malignant HT29 cells were present. RPMI medium contains 208 μM cystine, which can react with H_2_S to form cysteine and cysteine persulfide and, subsequently, other sulfane sulfur species. Indeed, cysteine and sulfane sulfur accumulation were observed within 30 min. In the presence of cells, the cysteine concentration was maintained at ~60 μM in 4 h, whereas it decreased to almost zero in culture medium alone over the same period (Fig. 2*B*). Of interest, in the absence of sulfide, cellular metabolism also contributed to a gradual increase in extracellular cysteine levels, although with different kinetics compared with cultured cells exposed to sulfide.

The sulfane sulfur concentration in the culture medium alone decreased more slowly to ~30 μM in 4 h (Fig. 2*C*). In the presence of cells, the sulfane sulfur levels also increased upon addition of Na_2_S and decreased more sharply to ~10 μM in 4 h. The concentration of the sulfane sulfur pool is higher than cysteine, suggesting that it comprises a mixture of low-molecular-weight and protein (present in serum) derivatives. Sulfane sulfur accumulation was not observed in RPMI medium alone, *i.e.*, in the absence of Na_2_S and cells. Similar results were obtained with DMEM medium (not shown).

In comparison with culture dishes with large surface areas, sulfide was more stable in Eppendorf tubes and the kinetics of its disappearance were clearly distinguishable in the presence of control cells with a scrambled shRNA sequence (HT29^scr^) (Fig. 2*D*). Cellular sulfide clearance was paralleled by the appearance of the oxidation product, thiosulfate (Fig. 2*E*). The 2:1 stoichiometry between sulfide and thiosulfate indicated that thiosulfate was the terminal oxidation product of HT29 cells under our experimental conditions.

### Sulfide stimulates glucose consumption and lactate production

**Figure 3:**
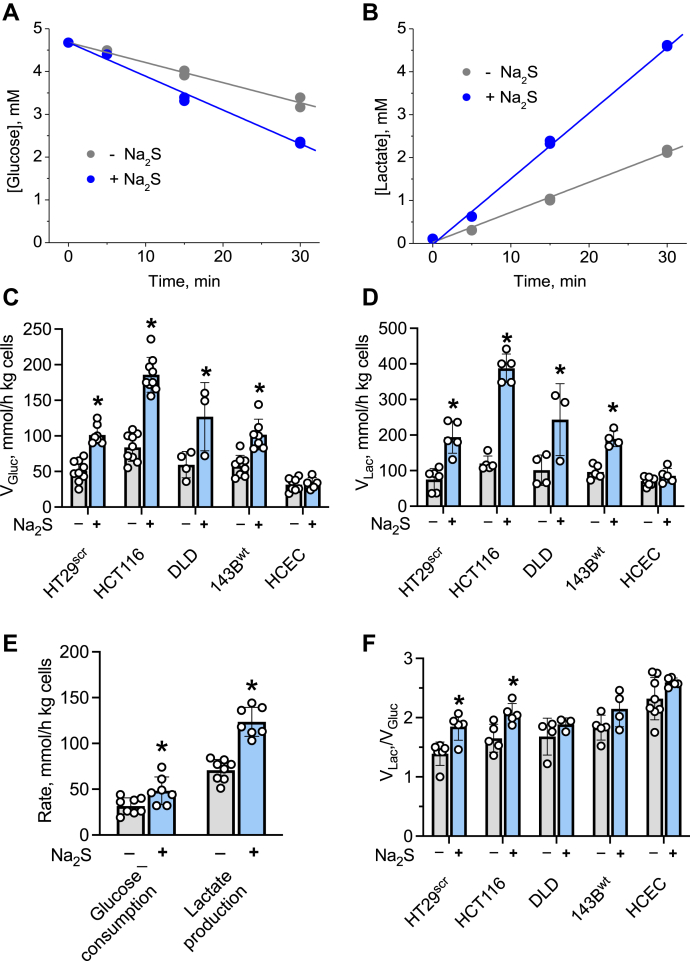
**Sulfide enhances aerobic glycolysis.** Kinetics of glucose consumption (*A*) and lactate accumulation (*B*) in 5% suspension (^w^/_v_) of HT29^scr^ cells in modified PBS (DPBS) in the absence (*gray*) or presence (*blue*) of 100 M Na_2_S. The data are representative of five independent experiments, each with technical duplicates. *C* and *D*, Na_2_S (100 μM) increases glucose consumption (*C*) and lactate production (*D*) rates in all but HCEC cell lines in 5% suspensions (^w^/_v_) in DPBS. *E*, at a higher concentration of Na_2_S (200 μM), increased glucose consumption and lactate production are also observed in HCEC cells. *F*, the lactate:glucose ratio obtained in the experiments is presented in *C* and *D*. The error bars represent the SD on the mean of 3 to 9 experiments. ∗Indicates statistically significant difference from control cells (*p* < 0.05).

The effect of H_2_S on the kinetics of glucose utilization was examined in 5% (^w^/_v_) suspension cultures in modified PBS (DPBS). Cells transfected with a scrambled shRNA sequence (HT29^scr^) exhibited enhanced glucose consumption and lactate production in response to H_2_S (100 μM) (Fig. 3, *A* and *B*). To assess whether stimulation of aerobic glycolysis was a general response to sulfide, four other cell lines were examined. A ~2- to 3-fold enhancement of glucose consumption and lactate production rates was observed at 100 μM sulfide in other malignant cell lines (HCT116, DLD-1, and 143B) as well (Fig. 3, *C* and *D*). No further stimulation of glycolysis was observed above this sulfide concentration as discussed later. In contrast, the nonmalignant HCEC cells did not increase aerobic glycolysis in response to 100 μM sulfide (Fig. 3, *C* and *D*). However, at a higher sulfide concentration (200 μM), a 1.6-fold activation of glycolysis was observed in HCEC cells (Fig. 3*E*).

Sulfide-induced activation of glycolysis increased the ratio of lactate produced to glucose consumed to the limiting value of two in HT29 and HCT116 cells (Fig. 3*F*). In DLD, 143B, and HCEC cells, the ratio was ~2 even in the absence of sulfide. Therefore, sulfide enhanced the rate of glucose oxidation in all the malignant cell lines tested here, whereas it increased the coupling between glucose consumed and lactate produced in HT29 and HCT116 cells, where the scope for further diverting pyruvate formed from glucose to lactate existed.

### SQOR knockdown sensitizes cells to activation of aerobic glycolysis by sulfide

We examined how enzymes in the mitochondrial sulfide oxidation pathway modulate sulfide clearance and the cytosolic response to sulfide, *i.e.*, enhanced aerobic glycolysis. For this, the first two pathway enzymes were knocked down in HT29 cells; SQOR as described previously (31) and ETHE1 (Fig. 2*F*). H_2_S clearance by control (HT29^scr^) and ETHE1 knockdown cells were indistinguishable while SQOR knockdown retarded sulfide clearance and thiosulfate production (Fig. 2, *B* and *C*).

**Figure 4:**
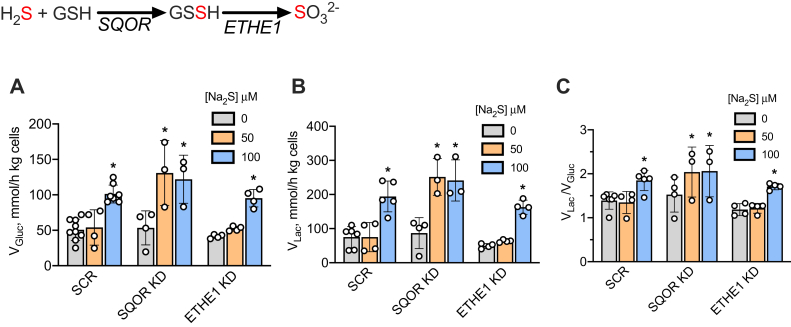
**Effects of the sulfide oxidation pathway on glucose metabolism.***A*–*C*, the rate of glucose consumption (*A*), lactate production (*B*), and the lactate:glucose ratio (*C*) in HT29^scr^ control SQOR or ETHE1 knockdown cells, incubated as 5% (^w^/_v_) suspensions in DPBS in the absence or presence of the indicated concentration of Na_2_S. The reactions catalyzed by SQOR and ETHE1 are shown above *A*. The error bars represent the SD on the mean of 3 to 9 independent experiments. ∗Indicates statistically significant difference from controls (lacking Na_2_S) (*p* < 0.05).

SQOR knockdown cells exhibited an increased sensitivity to H_2_S, and full activation of aerobic glycolysis was observed at 50 μM H_2_S (Fig. 4*B*). In contrast, a similar increase in the rate of aerobic glycolysis was observed only at 100 μM sulfide in HT29^scr^ control and ETHE1 knockdown cells. Corresponding changes were observed in the lactate:glucose ratio (Fig. 4*C*).

### Sulfide does not affect glucose flux through the oxidative pentose phosphate pathway or the tricarboxylic acid cycle

**Figure 5:**
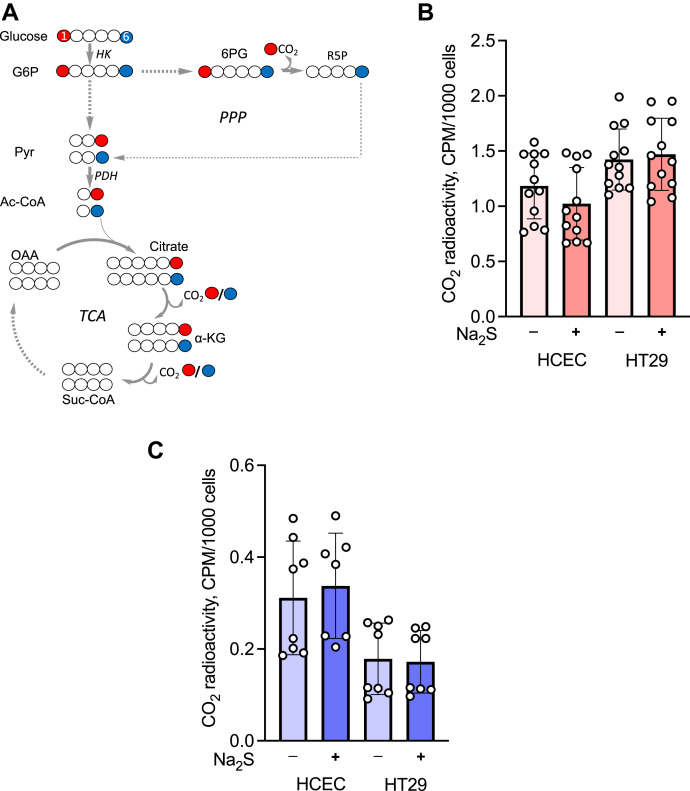
**Sulfide does not affect the oxidative pentose phosphate pathway or the TCA cycle.***A*, CO_2_ formation traced from glucose C1 (*red*) and C6 (*blue*). HK, PDH, PPP, and TCA denote hexokinase, pyruvate dehydrogenase, pentose phosphate pathway, and tricarboxylic acid cycle, respectively. G6P, Pyr, 6PG, R5P, OAA, and Ac-CoA denote glucose 6-phosphate, pyruvate, 6-phosphogluconate, ribose 5-phosphate, oxaloacetate, and acetyl-CoA. *B* and *C*, radiolabeled CO_2_ generation by HCEC or HT29 cells from [1-^14^C]-glucose (*B*) or [6-^14^C]-glucose (*C*) ± 100 μM Na_2_S over 4 h. The error bars represent the SD on the mean of two (*C*) or three (*B*) independent experiments each performed in quadruplicate.

Since H_2_S increases the glycolytic rate and reportedly increases the activity of glucose 6-phosphate dehydrogenase in a rat cardiomyoblast cell line (32), we examined whether it affects the pentose phosphate pathway and/or the tricarboxylic acid (TCA) cycle under our experimental conditions. As described previously (31), we used two bolus additions of sulfide separated by 3 h to compensate for its rapid disappearance from cell culture medium. The effect of sulfide on central carbon metabolism was monitored by measuring the formation of [^14^C]-CO_2_ from [1-^14^C]-glucose or [6-^14^C]-glucose (Fig. 5*A*). Metabolism of [1-^14^C]-glucose results in radiolabeled CO_2_ from both the oxidative pentose phosphate pathway and the TCA cycle, whereas [6-^14^C]-glucose liberates ^14^CO_2_ only *via* the TCA cycle. H_2_S did not change the extent of labeled CO_2_ from either [1-^14^C]- or [6-^14^C]-glucose in nonmalignant HCEC and malignant HT29 colonocytes, respectively (Fig. 5, *B* and *C*). Approximately 3.5-fold higher ^14^CO_2_ was released from C1 *versus* C6 glucose in HCEC cells and ~9-fold more in HT29 cells, indicating that the oxidative pentose phosphate pathway rather than the TCA cycle is the major source of CO_2_ in both these cell lines.

### Perturbations in mitochondrial redox status inhibit sulfide-activated aerobic glycolysis

**Figure 6:**
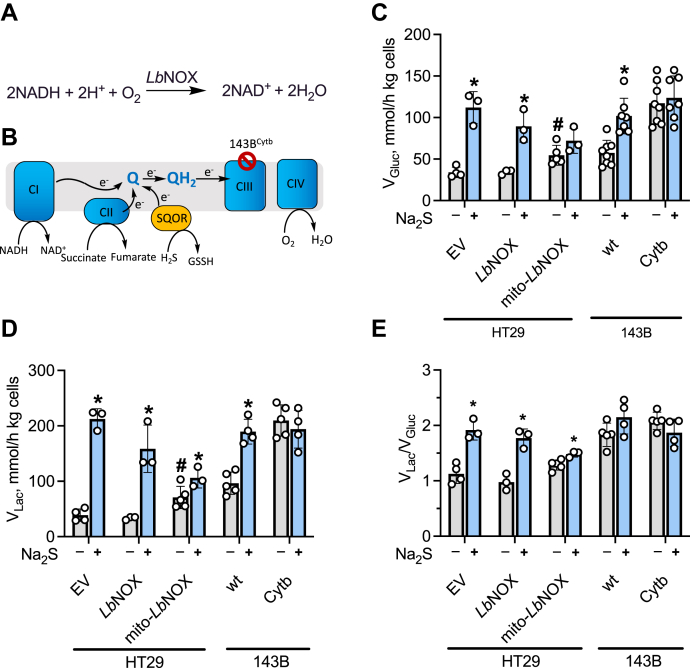
**Involvement of the mitochondrial NADH pool in signaling sulfide effects on glycolysis.***A*, the reaction catalyzed by *Lb*NOX. *B*, the electron transport chain where CI–CIV denote complexes I–IV and QH_2_ and Q denote reduced and oxidized CoQ. *C*–*E*, the effect of Na_2_S (100 μM) on the rates of glucose consumption (*C*) and lactate production (*D*), and the lactate:glucose ratio (*E*) in HT29 cells expressing empty vector (EV) or *Lb*NOX (cytoplasmic or mitochondrial) and in wildtype (143B^wt^) or mutant (143B^Cytb^) 143B cells. ∗Indicates statistically significant difference from the corresponding (-Na_2_S) controls, *p* < 0.05. ^#^Indicates statistically significant difference from empty vector control, *p* < 0.03. The error bars represent the SD on the mean of 3 to 8 independent experiments.

To assess the role of the mitochondrial *versus* cytoplasmic redox status on sulfide-stimulated aerobic glycolysis, two approaches were employed. First, HT29 cells expressing cytoplasmic or mitochondrial *Lb*NOX, the water-forming NADH oxidase from *Lactobacillus brevis* (33), were used (Fig. S1). In *Lb*NOX-expressing cells, the NADH/NAD^+^ ratio undergoes an oxidative shift in a compartment-specific manner (Fig. 6*A*). Second, 143B cybrids expressing either wildtype (143B^wt^) or mutant (143B^Cytb^) mitochondria (*i.e.*, lacking a functional complex III due to a frameshift mutation in cytochrome b (34)) were employed (Fig. 6*B*). In these cells, the mitochondrial pyridine nucleotide and CoQ pools are expected to be more reduced owing to a dysfunctional ETC.

Sulfide (100 μM) stimulated aerobic glycolysis in empty vector controls as well as in cells expressing cytoplasmic *Lb*NOX (Fig. 6, *C*–*E*), indicating that sulfide signaling is not mediated *via* the cytoplasmic pyridine nucleotide pool. In contrast, cells expressing mito-*Lb*NOX exhibited a 1.6-fold elevation in the basal glucose consumption rate, which was only slightly stimulated by 100 μM H_2_S (Fig. 6, *C*–*E*). These data indicate that mito-*Lb*NOX expressing cells activate aerobic glycolysis even in the absence of H_2_S to compensate for reduced mitochondrial ATP synthesis due to attenuated electron flux through complex I.

**Figure 7:**
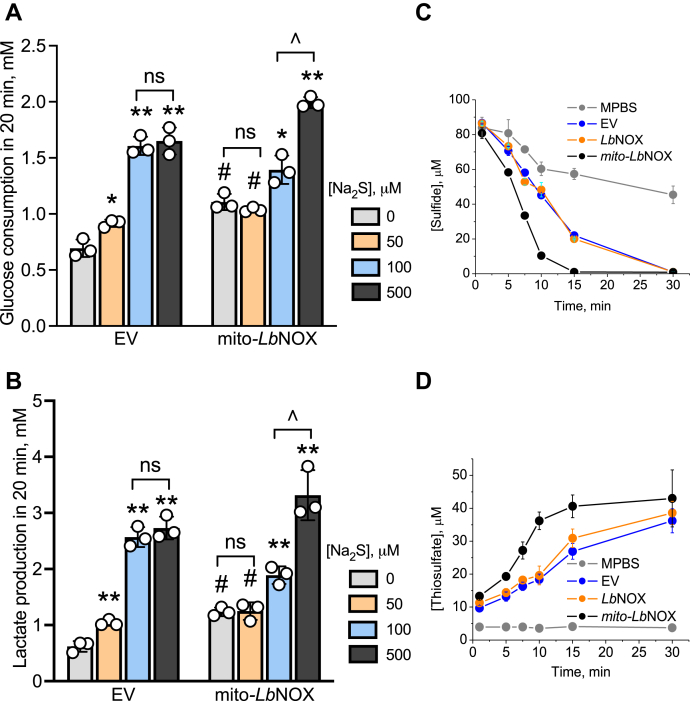
**Oxidation of the mitochondrial NADH pool decreases sensitivity to sulfide-mediated activation of glycolysis.***A* and *B*, glucose consumed in 20 min in 5% suspensions in modified PBS (DPBS) (^w^/_v_) of HT29 cells expressing an empty vector (*A*) or *mito*-*Lb*NOX (*B*) at different concentrations of Na_2_S. ∗,∗∗ Indicate a statistically significant difference from the corresponding control (minus Na_2_S) with *p* < 0.03 and *p* < 0.003, respectively. ^#^Indicates a statistically significant difference from the empty vector control, *p* < 0.003; ns is not significant. ˆIndicates a significant difference between the adjacent bars, *p* < 0.01. *C* and *D*, kinetics of sulfide disappearance (*C*) and thiosulfate formation (*D*) in DPBS alone or containing a 10% suspension (^w^/_v_) of HT29 cells expressing an empty vector or the cytoplasmic or mitochondrial forms of *Lb*NOX as indicated. Data represent the mean ± SD of three experiments.

Next, we examined the effect of H_2_S on glycolysis in mito-*Lb*NOX expressing cells in more detail. Unlike the empty vector control, maximal stimulation of aerobic glycolysis required a higher concentration of H_2_S (500 μM) confirming a lower sensitivity of these cells to H_2_S for eliciting a change in cytoplasmic energy metabolism (Fig. 7, *A* and *B*). The mito-*Lb*NOX expressing cells cleared H_2_S more rapidly than empty vector or cytoplasmic *Lb*NOX expressing cells, which was paralleled by an increased production of thiosulfate (Fig. 7, *C* and *D*). These data are consistent with reduced competition for the CoQ pool in mito-*Lb*NOX cells in which the mitochondrial NADH pool is more oxidized.

In comparison with 143B^wt^, the 143B^Cytb^ cybrids exhibited a higher basal glycolysis rate, and a 2:1 lactate production to glucose utilization ratio, even in the absence of sulfide, indicating that every equivalent of glucose entering the cell was metabolized to two equivalents of lactate (Fig. 6, *C*–*E*). Glycolysis was not further stimulated by H_2_S in these cells. Compared with 143B^wt^, suspensions of 143B^Cytb^ cybrids cleared H_2_S less efficiently and accumulated a lower concentration of thiosulfate (Fig. S2)

### Evaluation of the malate–aspartate shuttle in signaling sulfide-stimulated aerobic glycolysis

**Figure 8:**
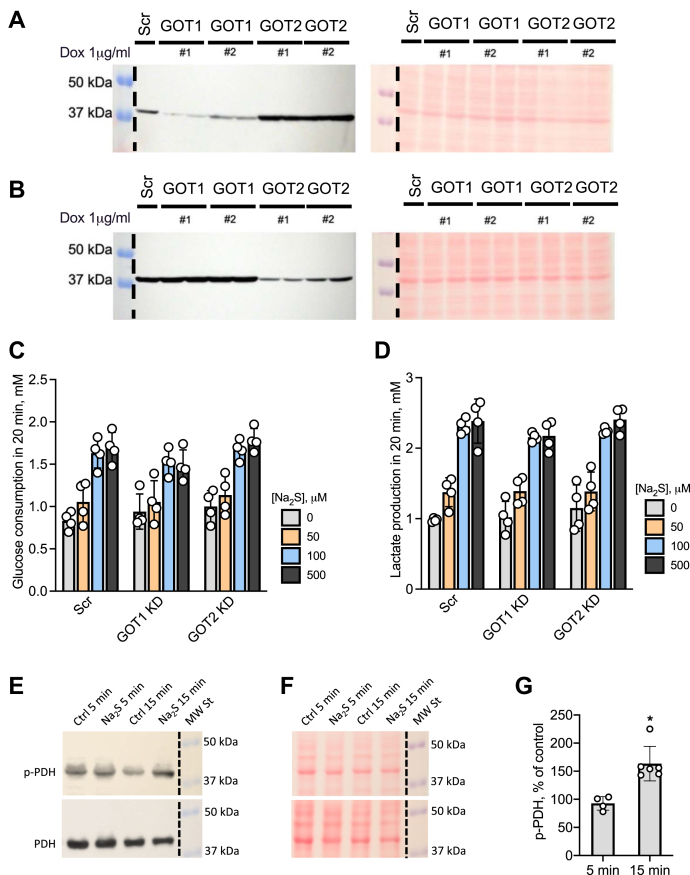
**Evaluation of signaling mechanisms for sulfide-stimulated aerobic glycolysis.***A* and *B*, expression level of GOT1 (*A*) and GOT2 (*B*) in GOT1 and GOT2 KD HT29 cells 96 h after treatment with 1 μg/ml doxycycline. Two shRNA sequences (#1 and 2) were used for targeting GOT1 and GOT2. Western blot analysis using anti-GOT1 antibodies (*left*) and Ponceau S staining of the same membrane showing equal loading (*right*). *C* and *D*, activation of glucose consumption (*C*) and lactate production (*D*) by the indicated concentrations of Na_2_S in HT29^scr^ or GOT1 or GOT2 knockdown cells. The concentration of glucose consumed and lactate produced in 20 min ± Na_2_S treatment. *E*, representative Western blot analysis of phosphorylated (*upper*) and total (l*ower*) pyruvate dehydrogenase in HT29^scr^ cells after 5 and 15 min ±100 μM Na_2_S. *F*, Ponceau staining of membranes in (*E*) demonstrating equal protein loading and transfer. *G*, quantitation of Western blot data for phosphorylated PDH shown in (*E*). The error represents SD on the mean of 4 to 6 experiments, ∗*p* < 0.005.

The malate–aspartate shuttle is a major conduit for moving electron equivalents between the cytoplasmic and mitochondrial compartments (Fig. 1). The role of this shuttle in sulfide signaling from the mitochondrion to the cytoplasm was examined by knocking down GOT1 and GOT2 in HT29 cells (Fig. 8, *A* and *B*). However, neither knockdown affected sulfide-stimulated aerobic glycolysis compared with control HT29^scr^ cells (Fig. 8, *C* and *D*).

Next, we examined whether sulfide stimulates phosphorylation of pyruvate dehydrogenase and thereby diverts pyruvate away from the TCA cycle. Although no change was discernable within 5 min of sulfide addition (100 μM), phosphorylation increased 164 ± 31% in 15 min (Fig. 8, *E* and *F*) and was sustained for at least another 15 min (not shown). A change in pyruvate dehydrogenase phosphorylation levels was not observed in HCT116 or 143B WT cells (Fig. S3), indicating that this is not a general mechanism for transducing sulfide effects from the mitochondrion to the cytoplasm.

### Sulfide perturbs adenine nucleotide pools

**Figure 9:**
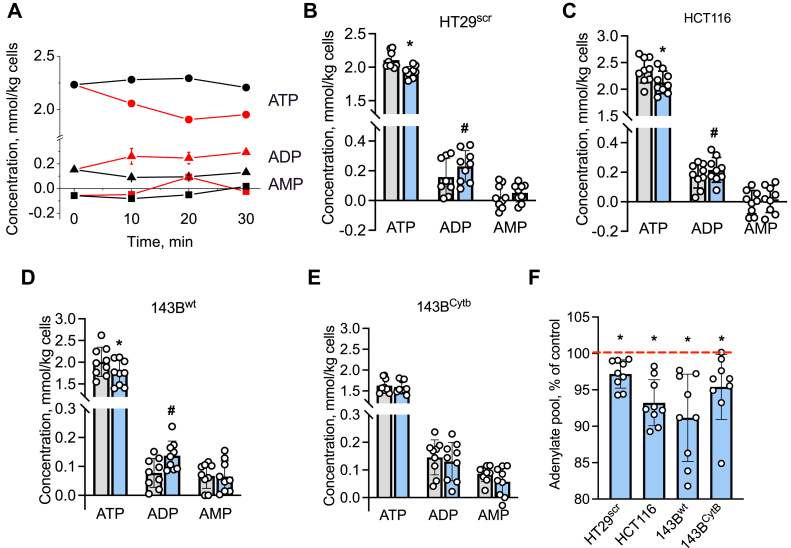
**Sulfide perturbs adenylate pools.***A*, time-dependent changes in ATP (*circles*), ADP (*triangles*), and AMP (*squares*) in a 5% suspension of HT29^scr^ cells in DPBS incubated with (*red*) or without Na_2_S (100 μM, *black*). Data are representative of three independent experiments. *B*–*E*, concentrations of ATP, ADP, and AMP in control (*gray bars*) and 100 μM Na_2_S (*blue bars*)-treated HT29^scr^ (*B*), HCT116 (*C*), 143B^wt^ (*D*), and 143B^Cytb^ (*E*) cells. Error bars represent the SD on the mean of three independent experiments, each performed in triplicate. ∗ and ^#^ Indicate statistically significant differences from control obtained using the paired *t* test, with *p* < 0.001 and *p* < 0.05, respectively. *F*, sulfide induced a decrease in the total adenine nucleotide pool (ATP + ADP + AMP) in the cell lines shown in *B*–*E*. The *red line* shows the control value in each cell line that is set at 100%. ∗Indicates statistically significant difference from control, *p* < 0.02.

Complex IV inhibition by sulfide is expected to reduce ATP synthesis *via* oxidative phosphorylation and perturb adenylate pools. Sulfide (100 μM) decreased ATP concentration ~8% to 12% in HT29^scr^ and HCT116 cells and 143B^wt^ cybrids (Fig. 9, *A*–*D*). A statistically significant increase in ADP was observed in the same cell lines. In comparison with 143B^WT^ cells, the 143B^Cytb^ cells showed lower total ATP and higher ADP, which did not respond to sulfide (Fig. 9*E*). AMP levels were low in most cell lines and reliable data could not be obtained owing to the high scatter. Compared with untreated controls, sulfide resulted in a statistically significant decrease in the total adenine nucleotide pool (*i.e.*, AMP + ADP + ATP) in each cell line (Fig. 9*F*).

## Discussion

One of the challenges in the H_2_S field is the varied sulfide concentrations that are used to elicit cellular effects and the lack of information on the lifetime of exogenously provided sulfide under different experimental conditions. Typically, concentrations varying from tens of micromolar to millimolar sulfide are used in cell culture experiments with high sulfide concentrations raising obvious questions about physiological relevance. The early studies using high sulfide concentrations occurred in the backdrop of a wide range of cellular concentration spanning 10^5^ orders of magnitude that had been reported in the literature (reviewed in (35)). In recent years, however, there has been a growing acceptance that steady-state H_2_S concentrations in mammalian cells are in the low nanomolar range (36, 37), although some cells, like colonocytes, have high exposure to sulfide derived from microbial metabolism that is estimated to be in the hundreds of micromolar to millimolar range (28, 29).

To provide a framework for deciphering sulfide effects under *ex vivo* conditions, we have examined the lifetime of exogenously supplied H_2_S in cell culture and cell suspension settings that are commonly used for metabolic studies. We found that H_2_S disappears rapidly under cell culture conditions (covered 6-cm plates, 37 °C) with a t^1^/_2_ of ~4 min regardless of whether or not cells are present (Fig. 2*A*). This abiotic loss is ascribed to the volatility of H_2_S as it is lost from PBS at an equivalent rate. However, a small fraction of H_2_S is trapped in a sulfane sulfur pool that is probably a mixture of low-molecular-weight (cysteine) persulfide and protein-bound species. The trapped sulfane sulfur pool, representing ~25% of the initially added sulfide (300 μM) at 1 h, decreases to ~10% at 4 h and could be a source of continued low sulfide exposure to cells in culture *via* sulfur transfer reactions as described by Equation 1.

(1) $$\text{RSSH}+{\text{R}}^{\prime }\text{SH}\to {\text{RSSR}}^{\prime }{+\text{H}}_{2}\text{S}$$

In suspension culture experiments performed in tubes with a smaller surface area than culture plates, the rate of abiotic sulfide loss is considerably lower and is visibly increased in the presence of cells, indicating metabolism (Fig. 2*D*). Sulfide metabolism in cell suspension was sensitive to the genetic ablation of SQOR, the first enzyme in the sulfide oxidation pathway (Fig. 1), but not to ETHE1. Of importance, sulfide was quantitatively converted to thiosulfate (Fig. 2*E*), indicating that thiosulfate could serve as a useful proxy for oxidative sulfide metabolism under these conditions.

The sensitivity of H_2_S-stimulated aerobic glycolysis to SQOR but not to ETHE1 ablation (Fig. 4) is consistent with the increased susceptibility of SQOR knockdown cells to complex IV inhibition (31). Upregulation of glucose consumption was observed in nonmalignant HCEC cells, which are dependent on growth factor–cued glucose uptake, as well as in malignant cells, which are not (Fig. 3, *C*–*F*). The nonmalignant HCEC cells, however, required higher concentrations of sulfide to elicit a similar metabolic response compared with the malignant cells (HT29, HCT116, DLD, and 143B). The mechanism by which aerobic glycolysis is dialed up in the cytoplasm in response to sulfide metabolism in the mitochondrion is not known. Several mechanisms have been proposed to explain the Warburg effect including hypoxia, decreased adenylate energy charge, loss of ETC function (*e.g.*, by inactivating mutations in complex II), accumulation of mitochondrial mutations, and activation of the rate-limiting steps in glycolysis (38). We examine the potential contributions of some of these mechanisms to sulfide-dependent retrograde signaling.

The theoretical maximum for the glucose consumed:lactate produced ratio is 2, *i.e.*, when every mole of glucose is converted to 2 mol of lactate. However, lactate can be derived from other sources as well, notably glutamine, which was not present in the DPBS buffer used in suspension culture. Glucose labeling studies, however, revealed that sulfide did not increase the flux of glucose through the oxidative pentose phosphate pathway or through the oxidative TCA cycle (Fig. 5). Even in cell lines in which the glucose consumed:lactate produced ratio was unaffected (DLD, 143B^wt^) the rates of glucose consumption and lactate production were enhanced (Fig. 3).

At low concentrations, sulfide serves as a substrate entering the ETC at the level of complex III (Fig. 1). At higher concentrations, sulfide inhibits complex IV, which is predicted to lead to membrane depolarization and, consequently, to decreased ATP synthesis by complex V. Consistent with this proposal, a decrease in ATP and an increase in ADP was observed in response to sulfide in HT29, HCT116, and 143B^wt^ cells (Fig. 9). In contrast, in 143B^Cytb^ cells in which the ETC is crippled, steady-state ATP levels were significantly lower and sulfide did not affect the adenylate pools. Decreased ATP levels in sulfide-treated cells are expected to stimulate a compensatory response by increasing glycolysis.

The increased glycolytic flux is balanced by increased lactate production, setting up a redox neutral cycle, which prevents buildup of cytosolic NADH. This is important in the setting of ETC inhibition by sulfide, which is expected to decrease the activity of the electrogenic glutamate–aspartate transporter, a component of the malate–aspartate shuttle (Fig. 1). Decreased malate–aspartate shuttling in sulfide-treated cells predicts decreased efflux of aspartate into the cytoplasm, explaining the partial aspartate auxotrophy that we had previously reported in sulfide-treated HT29 cells (31). The lack of effect of GOT1 or GOT2 knockdown on H_2_S-stimulated aerobic glycolysis (Fig. 8) is also consistent with a partial disabling of the malate–aspartate shuttle by sulfide.

Our study revealed a clear dependence on the mitochondrial but not the cytoplasmic NAD^+^/NADH ratio for signaling by sulfide. We had previously reported that the NAD^+^/NADH ratio is ~30% lower in sulfide-treated HT29 and HCEC cells, indicating an overall reductive shift in the pyridine nucleotide pool, which was further exacerbated in SQOR knockdown cells (31). Using the genetically encoded *Lb*NOX tool (33, 39), we could perturb the pyridine nucleotide pool in a compartment-specific manner. Our data clearly delineated a role for the mitochondrial NADH pool by revealing a blunted response to sulfide stimulation of aerobic glycolysis in cells expressing mito-*Lb*NOX (Fig. 6). In mito-*Lb*NOX expressing cells in which there is reduced competition for the CoQ pool from complex I, sulfide was cleared more rapidly, forming thiosulfate (Fig. 7).

In conclusion, we have demonstrated that the dual targeting of mitochondrial and cytosolic energy metabolism by sulfide involves its interaction with the ETC. The reductive shift in the mitochondrial NAD^+^/NADH pool is an important component in the sulfide signaling pathway, which leads to enhanced aerobic glycolysis. The capacity of sulfide to engage in metabolic signaling by tuning the ETC might be important for proliferation of normal and malignant cells, which rewire energy metabolism from oxidative phosphorylation to aerobic glycolysis to balance the energy and anabolic demands of cell division (2).

## Experimental procedures

### Materials

Na_2_S nonahydrate (99.99% purity), glucose, hydrocortisone, insulin, apo-transferrin, sodium selenite, sodium orthovanadate, uridine (cell culture grade), protease inhibitor cocktail for mammalian tissue extract, puromycin (Sigma P8833), and RIPA buffer were from Sigma. Monobromobimane (FluoroPure grade) was from Molecular Probes. [1-^14^C]-glucose (56.5 mCi/mmol), [6-^14^C]-glucose (60.3 mCi/mmol), and [U-^14^C]-glutamine (281.0 mCi/mmol) were from PerkinElmer. Cell culture media (DMEM with 4.5 g/l glucose, glutamine, and 110 mg/l sodium pyruvate [Cat. # 11995], RPMI 1640 with glutamine [Cat. # 11875], 199 [Cat. # 11150]), fetal bovine serum (FBS) (Cat. # 10437), penicillin/streptomycin mixture (Cat. # 15140), PBS (Cat. # 10010), and DPBS (Cat. # 14040) were from Gibco.

### Cell culture

Human colon cancer cell lines HT29, HCT116, and DLD-1 were obtained from American Type Culture Collection. The human osteosarcoma 143B^WT^ and 143B^Cytb^ cybrids were a generous gift from Dr Matthew Vander Heiden (MIT), and the nonmalignant human colon cell line HCEC was obtained from Dr Eric Fearon (University of Michigan).

HCT116 and DLD-1 cells were cultured in DMEM supplemented with 10% FBS, 100 units/ml penicillin, and 100 μg/ml streptomycin. 143B^WT^ and 143B^Cytb^ cybrids were cultured in the same medium except that 0.1 mg/ml uridine was also added. HT29 cells were cultured in RPMI 1640 medium supplemented with 10% FBS, 100 units/ml penicillin, 100 μg/ml streptomycin. The construction of HT29^scr^ and SQOR knocked down cell lines was described (31), and these cells were cultured as described above for HT29 except that the culture medium was supplemented with 1 μg/ml puromycin (Sigma).

With the exception of HCEC cells, all others were grown in humidified cell culture incubators at 37 °C containing a 5% CO_2_ atmosphere. HCEC cells were cultured in a mixture of DMEM and medium 199 (4:1 v/v), supplemented with 100 units/ml penicillin, 100 μg/ml streptomycin, 20 ng/ml human recombinant epidermal growth factor (Gibco), 2% (^v^/_v_) cosmic calf serum (Hyclone), 1 μg/ml hydrocortisone, 10 μg/ml insulin, 2 μg/ml apo-transferrin and 5 nM sodium selenite, in a humidified hypoxic incubator with an atmosphere containing 2% O_2_, 5% CO_2_, and 93% N_2_ at 37 °C.

### Generation of ETHE1 and SQOR knockdown cells

ETHE1 knockdown was performed in HT29 cells as described for SQOR knockdown (31). Briefly, the pLKO.1 vector with ETHE1-targeting shRNA sequences was purchased from Sigma (clone ID: NM_014297.3-770s1c1 and NM_014297.3-646s1c1). An shRNA vector purchased from Sigma with a scrambled sequence was used as a negative control. ETHE1 expression was not observed with NM_014297.3-770s1c1 (HT29^ETHEkd1^), whereas with NM_014297.3-646s1c1 (HT29^ETHEkd2^) there was lower knockdown efficacy (Fig. 2*F*). Transfected cells were cultured as described above for HT29 cells except that the cell culture medium contained 1 μg/ml puromycin (Sigma).

### Stable expression of cytoplasmic and mitochondrial *Lb*NOX

The pINDUCER vectors containing *Lb*NOX and mito-*Lb*NOX were a gift from Dr Costas Lyssiotis (University of Michigan). Empty vector, *Lb*NOX and mito-*Lb*NOX lentiviral vectors were produced by the Vector Core (University of Michigan). Stable HT29 cell lines expressing the cytoplasmic or mitochondrially targeted *Lb*NOX were generated as follows. Briefly, HT29 cells (25,000 per well) were seeded in a 12-well plate with each well containing 1 ml of RPMI 1640 medium supplemented with 10% FBS, 100 units/ml penicillin, and 100 μg/ml streptomycin. The cells were grown for 24 h at 37 °C in a humidified incubator with a 5% CO_2_ atmosphere. Cells were transduced with optimized viral titer. After 24 h, the medium in the wells was replaced with fresh virus-free medium and incubation was continued for an additional 24 h. Then, the cells were selected using medium containing 500 μg/ml geneticin (Life Technologies, 10131035). It took ~2 to 3 weeks to obtain confluent cells selected for *Lb*NOX expression during which time RPMI 1640 medium supplemented with 10% FBS, 100 units/ml penicillin, 100 μg/ml streptomycin, and 300 μg/ml geneticin was used. Before the start of an experiment, cells were incubated for 24 h with 300 ng/ml doxycycline (Sigma, D3447) to induce *Lb*NOX expression.

*Lb*NOX expression was validated 24 h after induction with 300 ng/ml doxycycline. Then, the cells were washed twice with PBS and lysed in RIPA buffer with 10 μl/ml protease inhibitor cocktail to prepare mammalian cell extracts. Lysates were frozen and thawed three times and centrifuged for 5 min at 12,000*g* at 4 °C. The supernatant was collected and protein concentration was determined using the Bradford reagent (Bio-Rad). Protein samples were separated on a 10% SDS polyacrylamide gel and then transferred to a PVDF membrane. Since *Lb*NOX was expressed with a FLAG tag, an anti-FLAG antibody (Sigma, F1804) was used to probe for its expression. Membranes were incubated overnight at 4 °C with mouse anti-FLAG antibody at a dilution of 1:2000. Horseradish peroxidase–linked anti-mouse IgG secondary antibody (GE Healthcare, NA931V) was used at a dilution of 1:20,000. The membranes were developed using Kwik Quant Digital-ECL substrate (Kindle Biosciences), and images were collected using a KwikQuant imager (Kindle Biosciences).

### Generation of cells with GOT1 and GOT2 KD

TET-pLKO-shGOT1, TET-pLKO-shGOT2, and TET-pLKO-shNT viruses were generous gifts from Dr Costas Lyssiotis (University of Michigan). Viral transduction of HT29 cells was performed as described above with *Lb*NOX. The cells were routinely cultured in RPMI 1640 medium supplemented with 10% FBS, 100 units/ml penicillin, 100 μg/ml streptomycin, and 1 μg/ml puromycin. To obtain efficient knockdown, shRNA targeting GOT1 or GOT2 was induced by treating cells with 1 μg/ml doxycycline for 96 h.

GOT1 or GOT2 knockdown was validated in cells 96 h after induction with 1 μg/ml doxycycline. Western blot analysis was performed similarly as described above for *Lb*NOX. Primary rabbit antibodies against GOT1 (Proteintech, 14886-1-AP) and GOT 2 (Proteintech, 14800-1-AP) were used at a dilution of 1:2000. Horseradish peroxidase–linked anti-rabbit IgG secondary antibody (GE Healthcare, NA944V) was used at a dilution of 1:20,000.

### Kinetics of sulfide consumption under standard cell culture conditions

HT29^scr^ cells were seeded at a density of 5 × 10^6^ cells per 6-cm plate containing 4 ml of culture medium and incubated overnight at 37 °C. The next day, the medium was replaced with fresh medium and Na_2_S was added from a freshly prepared stock solution in water. Plates containing culture medium but lacking cells were used as controls. The kinetics of abiotic sulfide disappearance was monitored in (i) DMEM (ii) RPMI, and (iii) PBS. The DMEM and RPMI 1640 media were supplemented with 10% FBS, 100 units/ml penicillin, and 100 μg/ml streptomycin. Each plate was used to obtain a single time point. In each experiment, plates with cell culture medium alone (*i.e.*, lacking Na_2_S) were used as blanks.

Aliquots of cell culture medium or PBS were collected at the desired time. For sulfide, thiosulfate, and cysteine analysis (45 μl), samples were mixed with 2.5 μl of 1 M Tris base and frozen immediately on dry ice and stored at −80 °C until further use. Samples for sulfane sulfur analysis (400 μl) were frozen immediately on dry ice and stored at −80 °C until further use.

### Cell suspension experiments

The culture medium was aspirated from confluent cultures in 10-cm plates and cells were washed twice with cold PBS and treated with 0.05% trypsin (Gibco). Trypsinized cells were suspended in cell culture medium and centrifuged at 1600*g* for 5 min at 4 °C. The supernatant was aspirated, and the cell pellet was suspended in 1 ml of DPBS, prepared from DPBS containing 100 mg/l CaCl_2_ and 100 mg/l MgCl_2_•6H_2_O, by adding 5 mM glucose and 20 mM Hepes, pH 7.4. The suspension was centrifuged at 1600*g* for 5 min at 4 °C, and the cell pellet mass was measured in a tared sample tube after the supernatant was aspirated. The cell pellet was suspended in DPBS to make a 5% to 10% cell suspension (^w^/_v_) and incubated at 37 °C with shaking at 75 rpm. Na_2_S was added from a freshly prepared stock to obtain the desired concentration. The cell suspension occupied ~70% to 80% of the tube volume, and the head space was filled with air. Samples (45 μl) were collected at the indicated time, mixed with 2.5 μl of 1 M Tris base, and frozen immediately on dry ice. Sulfide and thiosulfate levels in the samples were analyzed by HPLC as described next.

### HPLC analysis of sulfur metabolites

For sulfide, thiosulfate, and cysteine analysis the frozen samples were derivatized with monobromobimane as follows. The samples (45 μl) were thawed and 2.5 μl of 60 mM monobromobimane in dimethyl sulfoxide was added, and the mixture was incubated in the dark at 25 °C for 10 min. Then, 100 μl metaphosphoric acid solution (16.8 mg/ml) was added, the mixture was vortexed, centrifuged at 13,000*g* for 5 min at 4 °C, and the supernatant was stored at −20 °C until use. After derivatization with monobromobimane the samples were protected from light. The derivatized samples were analyzed on a Zorbax Eclipse XDB-C18 column, 5 μm, 4.6 × 150 mm (Agilent) with ammonium acetate and methanol gradient as described (40). Solutions of known concentrations of Na_2_S, sodium thiosulfate, and cysteine were used for calibration.

### Sulfane sulfur analysis in cell culture medium

For analysis, the samples (400 μl) were thawed and the sulfane sulfur levels were measured using the cold cyanolysis method (41) as described (40). Briefly, the sample was mixed with 0.2 ml of a solution containing 62.5 mM potassium cyanide and 125 mM ammonium hydroxide, and the mixture was incubated for 45 min at 25 °C. Then, 0.6 ml of Goldstein’s reagent was added, the mixture was vortexed and centrifuged for 3 min at 13,000*g*, and the absorbance of the supernatant was measured at 460 nm. The absorbance of blank samples was subtracted from sulfide-treated and control samples. Goldstein’s reagent was prepared by dissolving 1.25 g of Fe(NO_3_)_3_•9H_2_O in 12.5 ml water, to which 13.1 ml concentrated HNO_3_ and water were added to a final volume of 50 ml. A calibration curve was prepared using known concentrations of sodium thiocyanate solutions.

### Glucose and lactate analysis

To measure concentrations of glucose and lactate in cell suspension, 100-μl sample aliquots were removed and mixed with 200 μl of 5% HClO_4_, vortexed, and stored at −20 °C until use. Samples were thawed, vortexed, and centrifuged at 13,000*g* for 5 min at 4 °C. The supernatant was collected and neutralized to pH ~7 with saturated K_2_CO_3_ solution. The concentrations of glucose and lactate in the neutralized supernatants were measured using D-GLUCOSE-HK kit (Megazyme) and L-Lactate assay kit (Cayman Chemical), respectively, as per the manufacturers’ protocols. The rates of glucose consumption and lactate production were estimated by linear fits of the experimental data using Origin 7.0 software.

In some experiments with *Lb*NOX-expressing cells and with glutamate oxoglutarate transaminase ½ (GOT1/2) knockdown cells, the rates of glucose consumption and lactate production were assessed at 20 min and the change in glucose or lactate concentrations over this time period was reported.

### Estimation of adenine nucleotide concentrations

The concentrations of ATP, ADP, and AMP were measured in the same cell suspension samples (fixed with 5% HCLO_4_) that were used for glucose and lactate analysis described above. The measurements were performed using a luciferase-based ATP/ADP/AMP Assay Kit (Biomedical Research Service & Clinical Application, University at Buffalo, Cat. # A-125) as per the manufacturer’s protocol.

### Western blot analysis of pyruvate dehydrogenase

For Western blot analysis of pyruvate dehydrogenase in suspension culture, 400-μl aliquots were collected, placed on ice for a few minutes, and then centrifuged at 1600*g* for 3 min at 4 °C. Then, 350 μl of supernatant was removed and the remainder of the tube’s content was mixed with 200 μl RIPA buffer containing 10 μl/ml protease inhibitor cocktail and 1 mM sodium orthovanadate. The mixture was stored at −20 °C.

At the time of analysis, the sample was thawed, vortexed, and centrifuged at 13,000*g* for 5 min at 4 °C. Protein concentration was measured in the supernatant using the Bradford reagent (Bio-Rad). Proteins were separated on a 10% SDS polyacrylamide gel and transferred to a PVDF membrane. Samples from each experiment were run on two gels and transferred to two membranes for analysis of total and phosphorylated pyruvate dehydrogenase, respectively. Membranes were incubated overnight at 4 °C with primary antibodies at a dilution of 1:1000. Total pyruvate dehydrogenase was detected using rabbit monoclonal antibodies (Cell Signaling Technology, C54G1). Phosphorylated pyruvate dehydrogenase was detected using rabbit polyclonal antibodies to pyruvate dehydrogenase E1-alpha subunit (phospho S293) from NOVUS (NB110-93479SS) or from Abcam (ab92696). The secondary antibody, horseradish peroxidase–linked anti-rabbit IgG from GE Healthcare (Cat # NA934V) used at a 1:50,000 dilution. The membranes were treated with KwikQuant Digital-ECL substrate (KwikQuant), and images were collected with a KwikQuant Imager and quantified using ImageJ software. Equal loading was verified by Ponceau S staining of membranes.

### CO_2_ generation from [^14^C]-glucose

HT29 or HCEC (7 × 10^5^ cells per well) were cultured in 12-well plates and incubated overnight under standard conditions described above for each cell line. Then, the medium was replaced (2 ml/well) with fresh RPMI 1640 medium containing Hepes buffer, supplemented with 10% FBS, 100 units/ml penicillin, and 100 μg/ml streptomycin (Gibco) with 0.1 μCi per ml of [1-^14^C]- or [6-^14^C]-glucose (PerkinElmer) with or without Na_2_S (100 μM). The wells were covered with pieces of Whatman filter paper (grade 1) soaked with 1 M NaOH, and the cells were incubated at 37 °C in an ambient atmosphere. After 3 h, the cells were treated with a second bolus of Na_2_S (100 μM) and incubated for an additional hour. The reaction was terminated by addition of 300 μl of 3 M HClO_4_ per well, and the and the plates were kept overnight at ambient temperature. The Whatman filter papers were removed the next day, and radioactivity was measured using a liquid scintillation counter.

## Data availability

All data are contained within the article and in the supplemental section.

## Supporting information

This article contains supporting information.

## Conflict of interest

R. B. is a paid member of the scientific advisory board of Apneo Therapeutics and owns equity in the company.
