# Supplementary material for: The mitochondrial NADH pool is involved in hydrogen sulfide signaling and stimulation of aerobic glycolysis
Source: J Biol Chem. 2021 Apr 30;296:100736. doi: 10.1016/j.jbc.2021.100736 (PMC8165552; doi:10.1016/j.jbc.2021.100736)
Supplement: Supplemental Figures S1–S3 [file mmc1.docx]

**The mitochondrial NADH pool is involved in hydrogen sulfide signaling and stimulation of aerobic glycolysis**

Victor Vitvitsky^1^, Roshan Kumar^1^, Marouane Libiad^1,†^, Allison Maebius^1^, Aaron P. Landry^1^, and Ruma Banerjee^1^*

^1^Department of Biological Chemistry, Michigan Medicine, University of Michigan, Ann Arbor, MI 48109

**Running Title**: Sulfide catabolism regulates aerobic glycolysis

*Corresponding Author: email: [rbanerje@umich.edu](mailto:rbanerje@umich.edu), Tel: 734-615-5238

**Table of Content**

Figure S1. Expression of *Lb*NOX and *mito-Lb*NOX in HT29 cells.

Figure S2. Sulfide oxidation by 143B^wt^ and 143B^Cytb^ cybrids.

Figure S3. Sulfide does not affect phosphorylation of pyruvate dehydrogenase in HCT116 and 143B^wt^ cells.


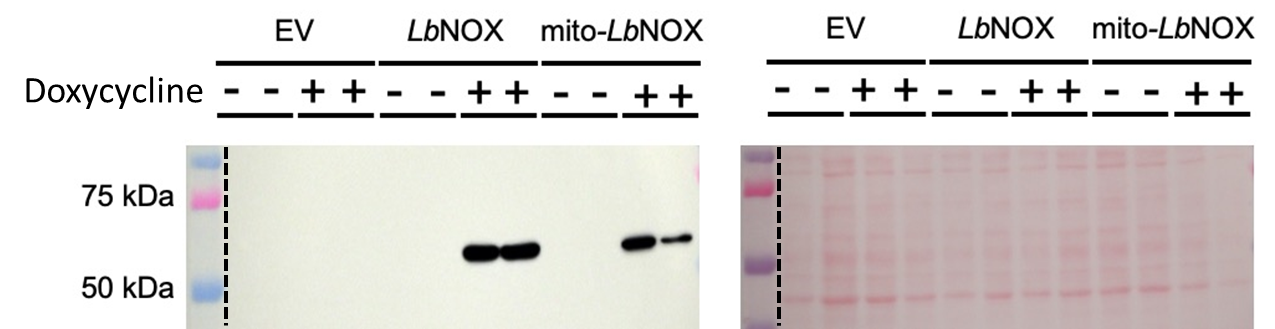


**Figure S1. Expression of *Lb*NOX and *mito-Lb*NOX in HT29 cells.** Western blot analysis of cell samples ± 300 ng/ml doxycycline after 24 h (*left*) and equal loading of the same membrane as visualized by Ponceau S staining (*right*).


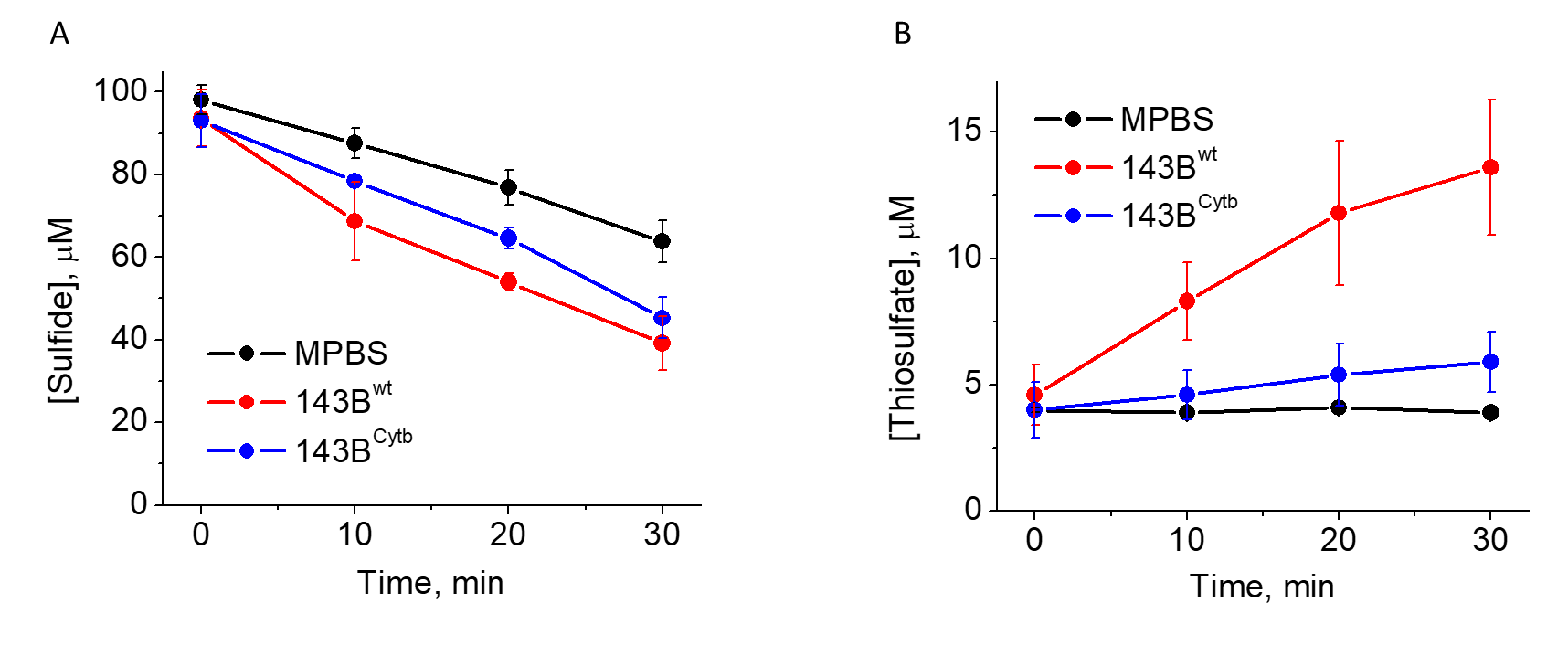


**Figure S2. Sulfide oxidation by 143B^wt^ and 143B^Cytb^ cybrids. A**. Kinetics of Na_2_S (100 µM) disappearance in MPBS (black) or in a 5% suspension (^w^/_v_ in MPBS) of 143^wt^ (red) or 143B^Cytb^ (blue) cybrids. **B**. Accumulation of thiosulfate in the same experiments as in (A). Data represent the mean ± SD of three experiments.


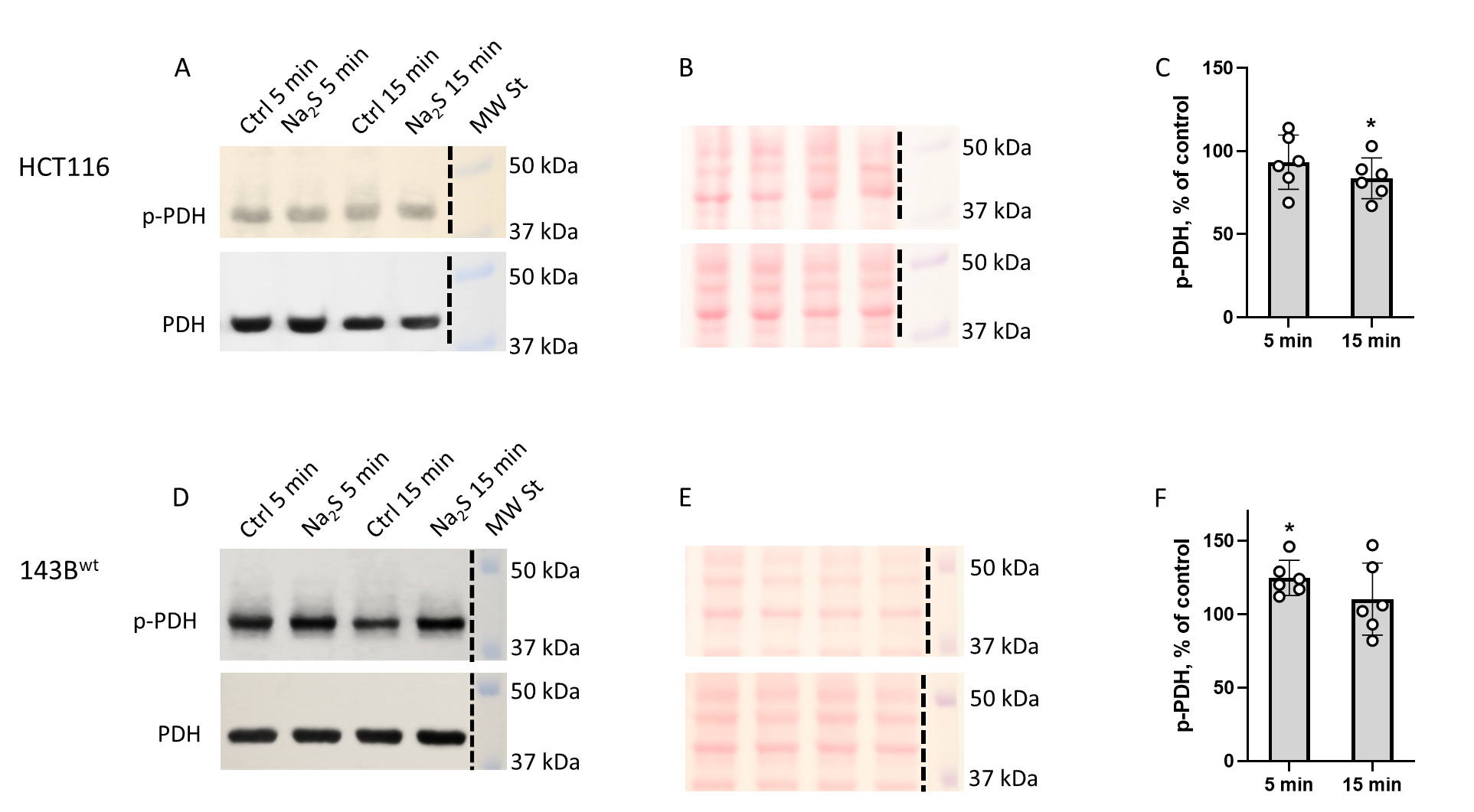


**Figure S3. Sulfide does not affect phosphorylation of pyruvate dehydrogenase in HCT116 and 143B^wt^ cells. A,D.** Western blot analysis of total (lower panel) and phosphorylated (upper panel) pyruvate dehydrogenase expression in HCT116 cells (A) or 143B^WT^ cells (D) in 5% cell suspension (^w^/_v_) in MPBS, 5 and 15 min after addition of 100 µM Na_2_S. **B,E.** Equal protein loading (30 µg/lane in B and 20 µg/lane in E) as shown by Ponceau S staining. **C,F.** Quantification of Western blot data for HCT116 cells (C) and 143B^wt^ cybrids (F). The errors represent SD on the mean of three independent experiments each run in duplicate. ^*^Indicates a statistically significant difference from control, p<0.005.
